# Axicon metalens for broadband light harvesting

**DOI:** 10.1515/nanoph-2023-0017

**Published:** 2023-03-08

**Authors:** Kai-Hao Chang, Yen-Chun Chen, Yo-Song Huang, Wei-Lun Hsu, Guo-Hao Lu, Chao-Feng Liu, Chun-Jen Weng, Yu-Hsin Lin, Che-Chin Chen, Chien-Chieh Lee, Yu-Chi Chang, Po-Hsiang Wang, Chih-Ming Wang

**Affiliations:** VisEra Technologies Company Limited, Hsinchu, 30078, Taiwan; Department of Optics and Photonics, National Central University, Taoyuan, 320371, Taiwan; National Applied Research Laboratories, Taiwan Instrument Research Institute, 20 R & D, Rd. VI, Hsinchu, 30076, Taiwan; Optical Science Center, National Central University, Taoyuan, 320371, Taiwan

**Keywords:** axicon, color router, hyperspectral imaging, imaging sensor, light harvest, metalens

## Abstract

In this study, an axicon metalens comprising a large central disc surrounded by nanoposts for energy harvesting in composite metal-oxide semiconductor sensors was designed, fabricated, and experimentally characterized. The main role of the central disc is focusing light; the nanoposts of various diameters deflect light to form a Bessel-like beam. The spatial distribution of the optical transmission was measured using micro-hyperspectral imaging. The axicon metalens concentrates the light to the sensitive area of the sensor and also harvests light from adjacent pixels. After adding an axicon metalens, the normalized peak transmission is up to 250% at *λ* = 700 nm as compared to a blank TiO_2_ film. The experimental results had fair agreement with the finite-difference-time-domain simulation. The ultra-broadband energy-harvesting performance of the sensor suggests that it could be applied in surveillance and Internet of Things applications.

## 1 Introduction

State-of-art imaging sensors must have high efficiency and high signal-to-noise ratios. Thus, the effective use of light energy is crucial in many aspects of optical systems, including focusing, filtering, detecting, and converting light in a specific frequency band. The performance of light-receiving devices, whether for visible, near-infrared (NIR), terahertz, or millimeter-wave light, may be limited by numerous factors, including the pixel size effect, the photoelectric conversion materials, and color coding [1]. These challenges and potential solutions are discussed in detail in this paper with a focus on methods relevant to visible and NIR sensors.

Simple light-receiving devices can only obtain intensity information; they require a bandpass color filter (either absorbing or reflecting) to extract color information in the visible range. For example, a Bayer filter pattern [2], which is a mosaic filter pattern comprising one part red (R), two parts green (G), and one part blue (B) color filters, for interpreting color information has a maximum efficiency of 25% for R and B pixels and 50% for G pixels; these efficiency levels depend on the overall percentage of the sensitive area [3]. Conventional spectral engineering materials, such as dye- or pigment-based absorbers and thin-film reflectors, filter light, resulting in the wastage of two-thirds of the incoming light at the stop bands. An imaging sensor’s quantum efficiency (QE) depends not only on the design of its optical elements but also on its optoelectronics conversion material. In the NIR range, the insufficient QE of silicon due to its low absorption coefficient limits its applications. Methodologies such as backside scattering [4] and inverted pyramid array structures [5] have been proposed to enhance the QE of photodiodes (PDs). However, these technologies require embedding additional nanostructures in PDs; the fabrication of such PDs by using standard complementary metal-oxide-semiconductor (CMOS) processes is challenging. Moreover, these embedded nanostructures occasionally result in high dark currents.

Recent advances in metasurface technology have enabled the nanoscale manipulation of light [6–8], providing a potential solution for the aforementioned challenges of producing a compact CMOS imaging sensor (CIS). Light harvesting and spectral routing by in metasurface-based CISs have been demonstrated to improve QE. Metasurfaces with spectral routing differ from conventional spectral filtering schemes; on these surfaces, incident light is sorted to a designated path on the basis of its wavelength. Hence, spectral routing techniques could surpass the efficiency of conventional dye- or pigment-based filter methods. For example, three-dimensional (3D) color routers have excellent light utilization and color collection efficiency for the RGB color model [9, 10]; these routers have multilayer stacked structures or 3D structures designed using the inverse design method [11–14]. However, 3D nanostructures are difficult to produce using conventional lithography. Recently, Zou et al. described a pixel-level metasurface-based Bayer-type color router based on a two-dimensional (2D) structure and developed using inverse design. The router achieved peak color collection efficiencies of 58%, 59%, and 49% for the R, G, and B spectral ranges, respectively, and had an average energy utilization efficiency of up to 84% for the visible range. This efficiency is approximately double that of a conventional pigment-based color filter [7]. Choo et al. realized a metaphotonic color-routing structure with QE and SNR improvements of approximately 20% and +1.22 dB, respectively, under low light (5 lux) [8]. Because color-routing metasurfaces are distant from the PDs, dark current, such as in [4, 5], did not occur.

Although algorithm-based optimization of color routers can be used to produce imaging devices with high performance, substantial computing resources are required for optimization. The number of generations required for optimization can be reduced if an appropriate initial condition is selected; various methods, such as the time-reversal method [15] and Green’s function method [16], have been proposed to achieve this goal.

Metasurface-based optical components can be combined to compose metalens. A single-layer metalens comprising various metasurface unit cells corresponding to different wavelengths achieved color routing and precise control of light focusing [6]. Similarly, Uenoyam et al. designed metalens to improve a photomultiplier tube’s response in the blue light range by focusing 404 nm incident light on the high-sensitivity region of a single photon avalanche diode, avoiding the low-sensitivity PD area [17]. This intuitive concept can also be applied to CISs because conventional CISs have a metal grid at the boundary of each pixel; this grid wastes some incident light. Moreover, the phase profile of metalens can be easily obtained using equal path theory [18]. Therefore, metalens can be designed more rapidly than other complex color-routing structures. However, Uenoyam et al. only optimized the light-harvesting metalens for a single wavelength; thus, its practical applicability was limited.

Currently, the color router is most for visible CIS. However, there are more applications in a low-light environment, such as night surveillance systems. Therefore, the RGB color router usually loses the energy in NIR. In this paper, we propose an axicon metalens comprising a large central disc surrounded by nanoposts for NIR harvesting. The central disc harvests input light and focus it to a small hot spot, and the nanoposts with various diameters deflect light to form a Bessel-like beam. The axicon metalens concentrates the light on the sensitive area and harvests some light from adjacent pixels, resulting in a higher QE. A transmission of over 250% can be achieved, and the transmission is greater than 100% in the 550–900 nm range. The experimental results agree with the 3D finite-difference-time-domain (FDTD) simulation. The performance of this ultra-broadband energy-sorting sensor suggests that it has potential for application as a CIS for surveillance and Internet of Things (IoT) applications.

## 2 Description of device

**Figure 1:**
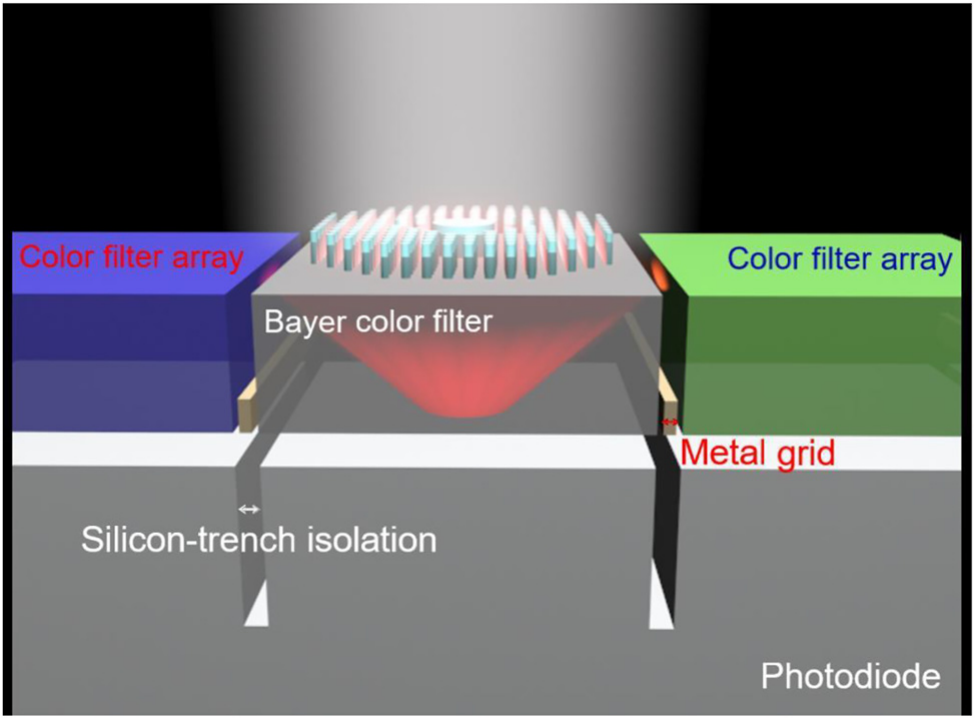
Schematic of the axicon metalens for CIS light harvesting. The top layer is a TiO_2_ pattern comprising a Bayer-type color filter. The axicon metalens is surrounded with RGB bandpass filters.

The proposed structure, an RGBW mosaic pattern, comprises a Bayer-type color filter with an inserted axicon metalens for broadband light harvesting to enhance the QE of a monochromatic PD (i.e., a W pixel). A schematic of the proposed structure is presented in Figure 1. Each RGB color filter and metalens for the W pixel are aligned and stacked on a square grid of CMOS PDs. The main difference between monochrome and color image sensors is that monochrome filters lack a color filter array (CFA) and IR-cut filter. Removal of these optical filters enables more photons to reach the bottom-most CIS, resulting in a higher QE. Consequently, W pixels have high performance in low light and at high frame rates.

The efficiency of an image sensor is affected by the total light reaching the sensor through the lens and its fill factor, which is the ratio of a pixel’s light-sensitive area to its total area. In conventional frontside-illuminated CIS, light must first pass through the CMOS before reaching the sensor, scattering the light and reducing the transmission efficiency. By contrast, the CMOS and photosensor layers are reversed in a backside-illuminated CIS, enabling the light to reach the photodiode directly without scattering and improving efficiency [19]. Despite these improvements, backside-illuminated CISs still have metal grids and silicon trenches at the boundary of each pixel for optical and electronic blocking, respectively, reducing the fill factor. A lower fill factor reduces sensor sensitivity and increases exposure times. However, the negative effect of these elements could be reduced by achieving a light path arrangement such that light was concentrated on the sensitive area, increasing the QE. More information regarding metalenses integrated on CISs is presented in the supporting information.

The metalens was produced using TiO_2_, which is CMOS-compatible, has a high refractive index, and has low optical loss. The metalens is an axicon that transforms a Gaussian beam into a Bessel-like beam distribution. Compared with Gaussian beams, Bessel beams have a greater depth of focus (DOF), increasing the fabrication tolerance related to the distance between the metalens and CMOS sensors. Moreover, the dispersion of the designed structure is small, facilitating broadband light harvesting.

**Figure 2:**
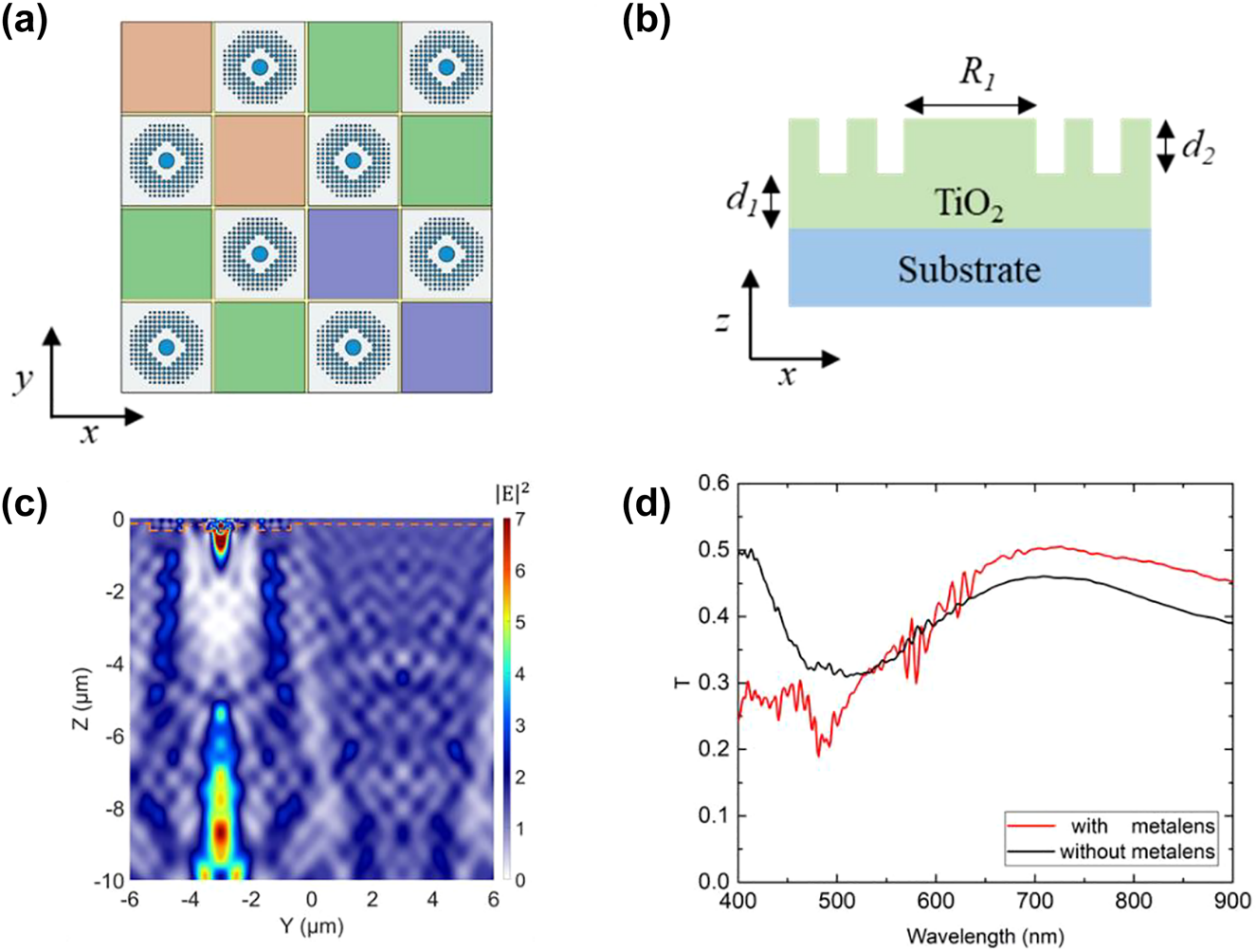
Schematic diagrams and simulation results of the axicon metalens. (a) Schematic of the Bayer RGBW CFA 2.0 layout with the inserted axicon metalens for W pixels. The pixel size of each mosaic is 6 × 6 μm. (b) Schematic of the proposed axicon metalens for broadband light-harvesting; *d*_1_ and *d*_2_ represent the height of the TiO_2_ nanopost and the thickness of the bottom TiO_2_ layer, respectively. (c) Electric field distribution at the *yz* plane for *λ* = 700 nm. (d) Simulated transmission spectrum of the Bayer RGBW CFA 2.0 layout with the axicon metalens inserted at W pixels. The black and red solid lines indicate the transmission at the RGB pixel (without the metalens) and at the W pixel (with the metalens), respectively.

The design and simulations were performed based on the FDTD method by using Lumerical, a commercial simulation tool [20]. The dielectric axicon metalens was simulated and optimized using a 3D model with periodic boundary conditions on its sides and perfectly matched layers on the top and bottom of the simulation domain. R, G, B, and W pixels (6 μm × 6 μm) were arranged in accordance with the RGBW CFA 2.0 [21, 22]; these pixels are presented in Figure 2(a). The 4 × 4 pattern CFA 2.0 comprises eight W pixels, four G pixels, two R pixels, and two B pixels and repeats across the entire image. The axicon metalens acts as the W pixel for broadband sensing. For night surveillance application, we enhance the transmission of NIR in W pixel by harvesting the NIR from neighboring pixels. In this CFA scheme, the W pixels comprise 50% of the entire domain in a checkerboard pattern surrounding each R, G, and B pixel; this W-pixel array improves sensitivity and resolution in low-light environments. Pixels must be small for high-resolution applications [23]; however, small pixels are ineffective in low-light applications. Therefore, the large pixel size of 6 μm × 6 μm was selected for improved sensitivity.

The axicon metalens comprises a central TiO_2_ disc surrounded by nanoposts with various diameters. The geometric parameters of the axicon metalens are presented in Figure 2(b). *R*_1_ denotes the diameter of the central disc; the diameters of the surrounding nanoposts are listed in the supporting information. *d*_1_ is the thickness of the axicon metalens (i.e., the thickness of the central disc and nanoposts), and *d*_2_ denotes the thickness of the bottom TiO_2_ layer. The axicon metalens has 4-fold rotational symmetry. Therefore, the optical response for normally incident light with *x*- and *y*-polarization is identical. We assumed that the input light is polarized at 45° with respect to the *x*-axis to simulate unpolarized light. The dispersion relation of TiO_2_ and silica substrate are from [24, 25], respectively. The TiO_2_ disc was regarded as the central disc of a binary-phase Fresnel lens. The refractive index of the thin-film TiO_2_ at the NIR spectral range is *n* = 2.4. The corresponding optical path with a disc thickness of 150 nm is 2.4 × 150 = 360 nm, corresponding to π phase retardation at *λ* = 720 nm.

The phase distribution of the nanoposts is similar to that of an axicon and plays a role in deflecting the exterior light to the sensitive area of the CIS. An axicon concentrates a planewave onto a line segment along the optical axis. The length of this line segment is the DOF. The input light can be gathered with a long DOF due to the phase distribution of the axicon. Typically, nanoposts of various diameters are used to achieve the phase distribution of a flat lens. However, the phase gradient at the center of the lens, especially for a lens with a small numerical aperture (NA), is miniscule. In this case, a large disc is sufficient to represent the requisite phase distribution, and fabricating a large disc is easier than fabricating nanopost arrays. Figure 2(c) presents a cross-section of the simulated electric field in the *yz* plane taken at the center of the axicon metalens for *λ* = 700 nm and reveals that the field can be focused from 7 to 10 μm. This distance corresponds to the typical distance from the air–CAF to the CAF–CMOS interface.

Figure 2(d) presents the transmission spectra at the W and RGB pixel positions (black and red solid lines, respectively), normalized to the total incident light power. The dielectric constant of the RGB pigment was not included in the FDTD simulation; therefore, the RGB pixels were assumed to be an optically transparent material, silica, for subsequent comparisons of simulations and measurements. Because the W and RGB pixels each occupy 50% of the total CFA array, their upper-limit transmissions are both 50%. Fabry–Perot resonance dips at *λ* = 550 nm and *λ* = 490 nm were observed for the RGB and W pixels, respectively. The TiO_2_ is assumed to be lossless. Therefore, any transmission less than 50% is due to reflection and scattering loss.

However, the transmission is greater than 50% at 700–720 nm, which is close to wavelength of maximum photon flux wavelength of solar. The transmission of the W pixels is approximately 8% higher than that of RGB pixels from *λ* = 600 nm to *λ* = 900 nm. This broadband enhancement in the R and NIR range is beneficial for surveillance applications.

## 3 Fabrication

**Figure 3:**
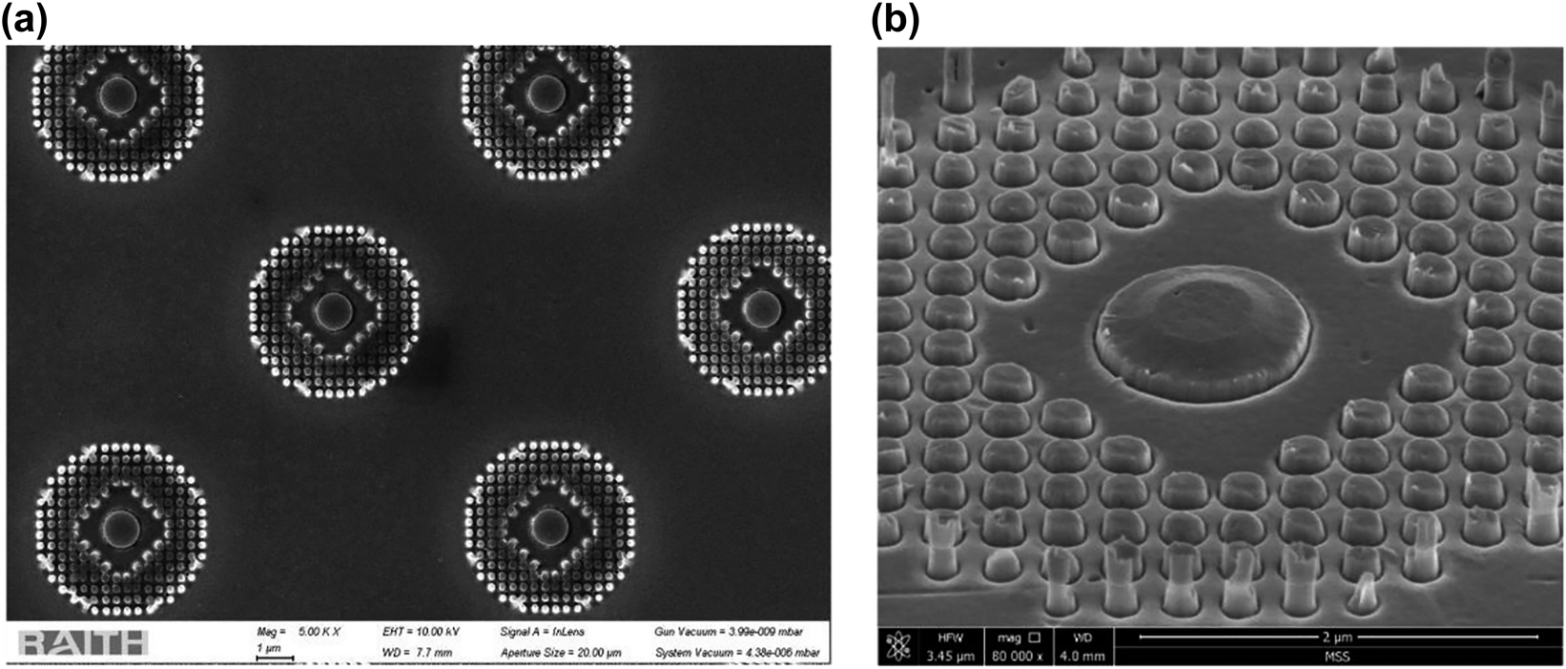
SEM images of the color-routing axicon metalens. (a) Front view, scale bar = 1 μm. (b) Glancing angle view, scale bar = 2 μm. The top layer TiO_2_ patterns are on a silica substrate with a thickness of 500 μm.

To verify the performance of the proposed axicon metalens experimentally and measure its optical properties, the axicon metalens was fabricated on an optically transparent fused-silica substrate. First, a TiO_2_ thin film with a thickness of 300 nm was deposited on a silica substrate by pulse-DC magnetron sputtering. Subsequently, the metalens pattern comprising a central disc and nanoposts was produced using electron-beam lithography (Raith Nanofabrication, Raith150 Two) on a spin-coated negative photoresist (Micro Resist technology, maN-2405). Finally, the metalens pattern was transferred to the TiO_2_ film by using a plasma dry etching process (Plasmalab System 100, Oxford Instruments) with mixed CF_4_ gas at 20 sccm and Ar gas at 10 sccm under inductively coupled plasma power of 1500 W and a bias power of 50 W. The etching depth was *d*_1_ = 150 nm. The thickness of the bottom TiO_2_ layer was *d*_2_ = 150 nm. Top-view and glancing-angle-view scanning electron microscope (SEM) images of the fabricated TiO_2_ metalens are presented in Figure 3(a) and (b), respectively.

## 4 Hyperspectral microscopic imaging

**Figure 4:**
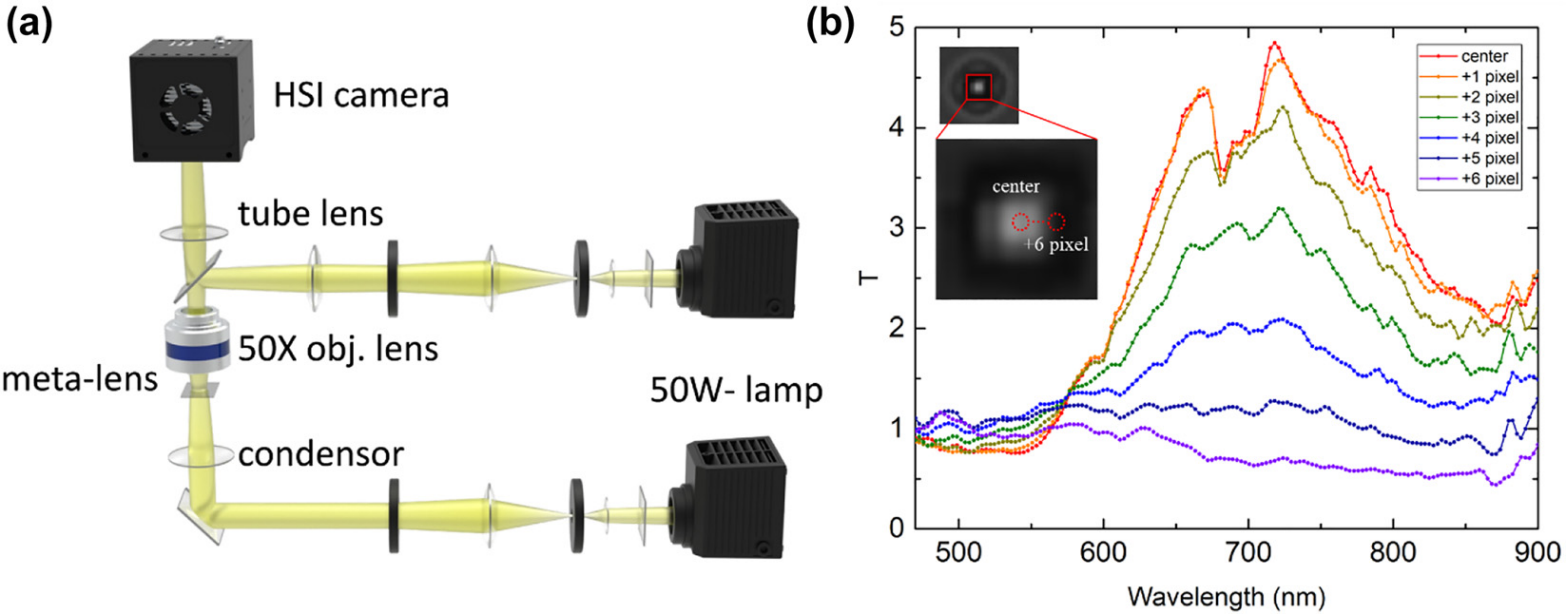
Optical measurement setup and transmission spectrum of axicon metalens. (a) Schematic of the μ-HSI apparatus with a 50× objective lens. Two 50 W halogen lamps were used for reflection imaging and transmission measurement. (b) Transmission spectra of the axicon metalens along the *x* direction; *x* = 0 indicates the center of the HSI lens; *x* = 6 indicates 6 pixels (on the HSI system) away from the center of the lens.

A micro-hyperspectral imaging (μ-HSI) system was used to measure the spectral response of the axicon metalens at a subpixel spatial resolution. The system comprised a conventional microscope and a hyperspectral imaging (HSI) camera [26]. Figure 4 depicts the setup for the μ-HSI measurements of the transmission of the metalens. First, a white light source illuminated the metalens from the top to the bottom to identify the surface of the metalens. After the metalens was vertically positioned, white light from a 50 W halogen lamp was passed through a relay lens, collimating lenses, and condenser (NA = 0.2) and then impinged on the metalens from the substrate side. The transmitted beam was collected by an achromatically corrected objective lens (50×, NA = 0.8) and then coupled into the HSI camera. The measuring wavelengths were 470–900 nm. An HSI camera, a 10 bit spectral image sensor with high spatial (2048 × 2048) and spectral (>150 bands) resolutions, was used to capture the transmitted images from the metalens. The pixel size of the HSI camera was 5.5 μm × 5.5 μm. An area of 54 × 54 pixels on the HSI camera was used for the μ-HSI measurements of the optical properties of each 6 μm × 6 μm metalens pixel at a digital resolution of 0.11 μm per HSI pixel in the object plane. According to the theory of diffraction, the diffraction-limited spot size is 1.22 *λ*/NA. If *λ* = 500 nm, the corresponding diffraction-limited spot size is 0.76 μm for NA = 0.8, indicating that the spatial resolution is limited by the diffraction but not by the HSI pixel size. Moreover, a manual *z*-axis stage with 1 μm resolution was used to move the metalens for scanning both in- and out-of-focus transmitted beam patterns.

Figure 4(b) presents the transmission spectrum of the axicon metalens measured at various pixels using the HSI system; *x* = 0 indicates the spectrum of the center of the metalens measured at the central HSI pixel. At the center of the metalens, the transmission is 5-fold greater than that of a blank TiO_2_ sample. At outer pixels, the transmission decreases without observable dispersion. A slight transmission peak exists at *x* = 5, corresponding to 0.5 μm in the object plane. Subsequently, the spectrum of the entire HSI image was measured; the results are discussed in the following section.

## 5 Spatial distribution and enhanced spectral response

**Figure 5:**
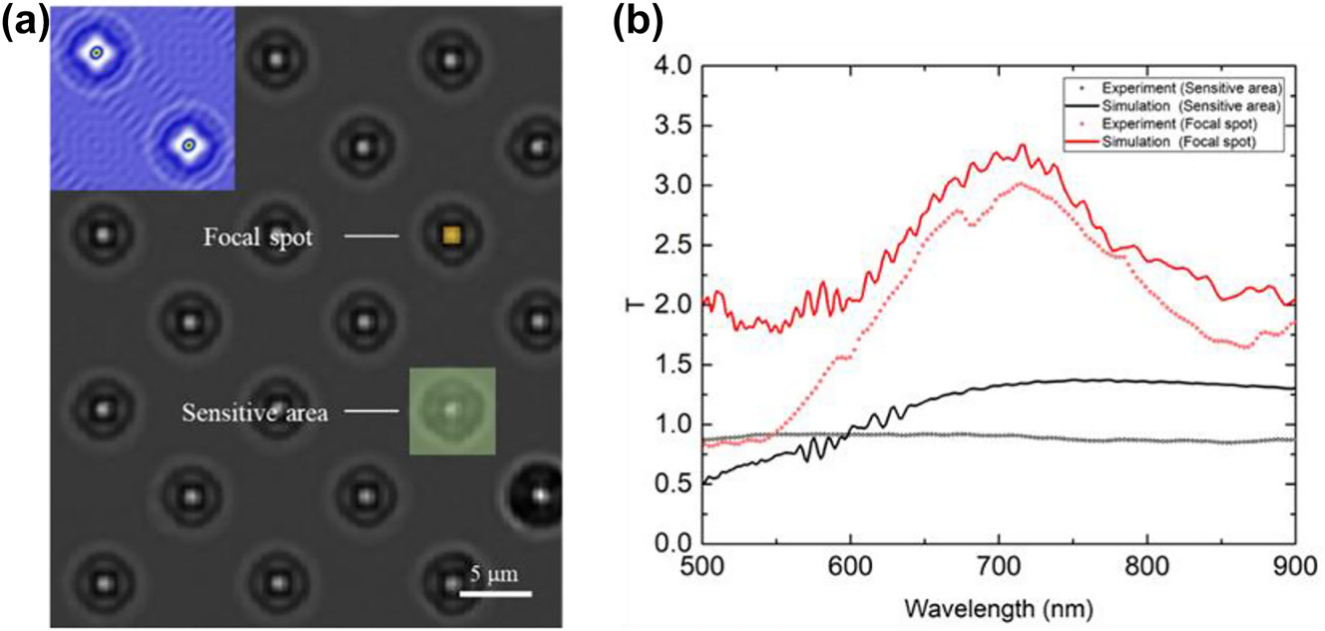
Transmission spectrum of axicon metalens with different measuring areas. (a) Measured optical microscope image of the axicon metalens array illuminated by collimated white light. The scale bar is shown in the image. Green and orange squares represent the collection area at the focal spot and at the sensitive area, respectively. The inset is the simulated intensity distribution at *λ* = 700 nm. (b) Transmission spectrum normalized to the transmission of a TiO_2_ film with a thickness of 150 nm. Red and black represent the transmission spectrum at the focal spot and at the sensitive area, respectively. Solid and dotted lines indicate the simulated and experimental results, respectively. The collection plane is 1 μm above the surface of the sample.

Figure 5(a) presents an optical microscope (OM) image of the metalens on a silica substrate. A focused hot spot was observed at the center of the metalens even for broadband unpolarized light. For reference, the inset of Figure 5(a) presents the simulated electric field intensity for *λ* = 700 nm. The input light was assumed to be linearly polarized with a 45° azimuth angle. The simulated intensity distribution is similar to that of the OM image. The orange and green squares represent the μ-HSI collection area at the focal spot and at the sensitive area, respectively. The orange square is 7 × 7 pixels on the HSI system, corresponding to an area of 0.77 μm × 0.77 μm on the sample. The green square is 53 × 53 pixels on the HSI camera, corresponding to an area of 5.8 μm × 5.8 μm on the sample. The location of the sensitive area was assumed to be similar to that a typical CIS fabricated by using a commercial KrF 248 scanner; that is, the linewidth of the silicon trench and the metal grid were assumed to be 200 nm.

As shown in Figure 5(b), the transmission spectrum at the focal spot and at the sensitive area was normalized to that of the TiO_2_ film away from the sample area. Red and black represent the transmission spectrum at the focal spot and at the sensitive area, respectively, and the solid and dotted lines represent the simulated and experimental results. The *z*-position of the collection plane was 1 μm above the sample surface. A transmission peak at *λ* = 700 nm was observed at the focal spot area that was 3-fold and 3.4-fold higher in the measurements and simulations than that of the flat TiO_2_ film with *d*_2_ = 150 nm. Moreover, the small peak at *λ* = 580 nm corresponds to the Fabry–Perot resonance at the silica/TiO_2_/air interfaces.

The measured and simulated transmission of the sensitive area was approximately constant at visible and NIR wavelengths; the average measured transmission was 86.87%. Differences between the experiment and simulation were attributed to scattering due to the roughness of the fabricated axicon metalens. Generally, for a typical CIS, transmission from the air to the CMOS PD is approximately 75%. The measured average transmission of 86.87% represents an improvement in the external QE of a backside-illuminated CIS; this external QE enhancement would be higher for a low-cost frontside-illuminated CIS.

## 6 Conclusions

An axicon metalens comprising a large central disc surrounded by nanoposts for CIS energy harvesting was designed, and the design was experimentally verified. The central disc plays a key role in focusing light; the nanoposts with various diameters deflect light to form a Bessel-like beam with a long DOF. The spatial and spectral responses of the axicon metalens were measured using μ-HSI. The axicon metalens concentrates the light on the sensitive area and harvests some light from adjacent pixels, resulting in a transmission of up to 300% at *λ* = 700 nm; this wavelength corresponds to the peak wavelength of sunlight. The experimental results had fair agreement with the FDTD simulation. At the hot spot, the transmission spectral range for 550–900 nm was greater than 100%. The high ultra-broadband energy-harvesting performance of the metalens suggests that it could be used in surveillance and IoT applicationse.
